# Mortification and Sloughing of a Part of the Intestine in Hernia

**Published:** 1833

**Authors:** 


### VII. Mortification and Sloughing of a part of the Intestine in Hernia. Recovery without an external aperture.

We are indebted to Dr. Silas Reed, now of this city, for the following fact.

Some years since, Dr. Joseph DeWolf, of Ravenna, in this state, was called to attend on Mr. McLaughlin of that place, laboring under strangulated inguinal hernia. All the ordinary methods of reducing it, having been tried without success, Dr. DeWolf performed the operation. The bowel was found in a state of gangrene. Five inches of it were removed; and the ends brought into apposition and secured by several ligatures. It was then returned and the external wound dressed in the usual way. It healed up, and the patient recovered. We hope in our next number to be able to give a detailed account of this interesting case.

